# The role of platelet-rich plasma therapy in refractory folliculitis decalvans

**DOI:** 10.1016/j.jdcr.2021.04.008

**Published:** 2021-04-24

**Authors:** Susie Suh, Cristina Nguyen, Ludan Zhao, Natasha Atanaskova Mesinkovska

**Affiliations:** a Department of Dermatology, University of California, Irvine, California; b Case Western Reserve University School of Medicine, Cleveland, Ohio

**Keywords:** cicatricial alopecia, folliculitis decalvans, platelet-rich plasma, PRP, scarring alopecia, treatment, FD, folliculitis decalvans, PRP, platelet-rich plasma, TAC, triamcinolone

## Introduction

Folliculitis decalvans (FD) is a rare, chronic primary scarring alopecia that classically presents with perifollicular pustules, scaling, crusting, and tufted hair in the affected areas of the scalp, accompanied by pruritus or pain.^1^ The cause of FD remains unclear; however, on histopathology, it presents as a predominantly neutrophilic inflammatory process targeting hair follicles, resulting in irreversible damage and permanent hair loss.^1^

Currently, there is no cure for FD. Most treatment regimens are directed at controlling the scalp-associated microbes with antibiotics and antiseptics, managing the inflammation, and preventing further hair loss with immunomodulators.^2^ Recent case reports have suggested that autologous platelet-rich plasma (PRP) can be a promising adjunct therapy for the treatment of lymphocytic scarring alopecia.^3^^,^^4^ However, there are no data on the efficacy of PRP in neutrophilic scarring alopecias, such as FD.

Here, we present 2 patients with long-standing, refractory FD who experienced stability in hair loss and significant symptomatic improvement in inflammation with concurrent PRP treatments. The severity of scalp inflammation was graded (Table I) by a single dermatologist (NAM) during each clinical visit.

**Table I:** Grading scale of disease severity

Score	Degree of erythema/scale/pustules
0	No involvement
1+	Mild
2+	Moderate
3+	Severe

## Case 1

**Fig 1:**
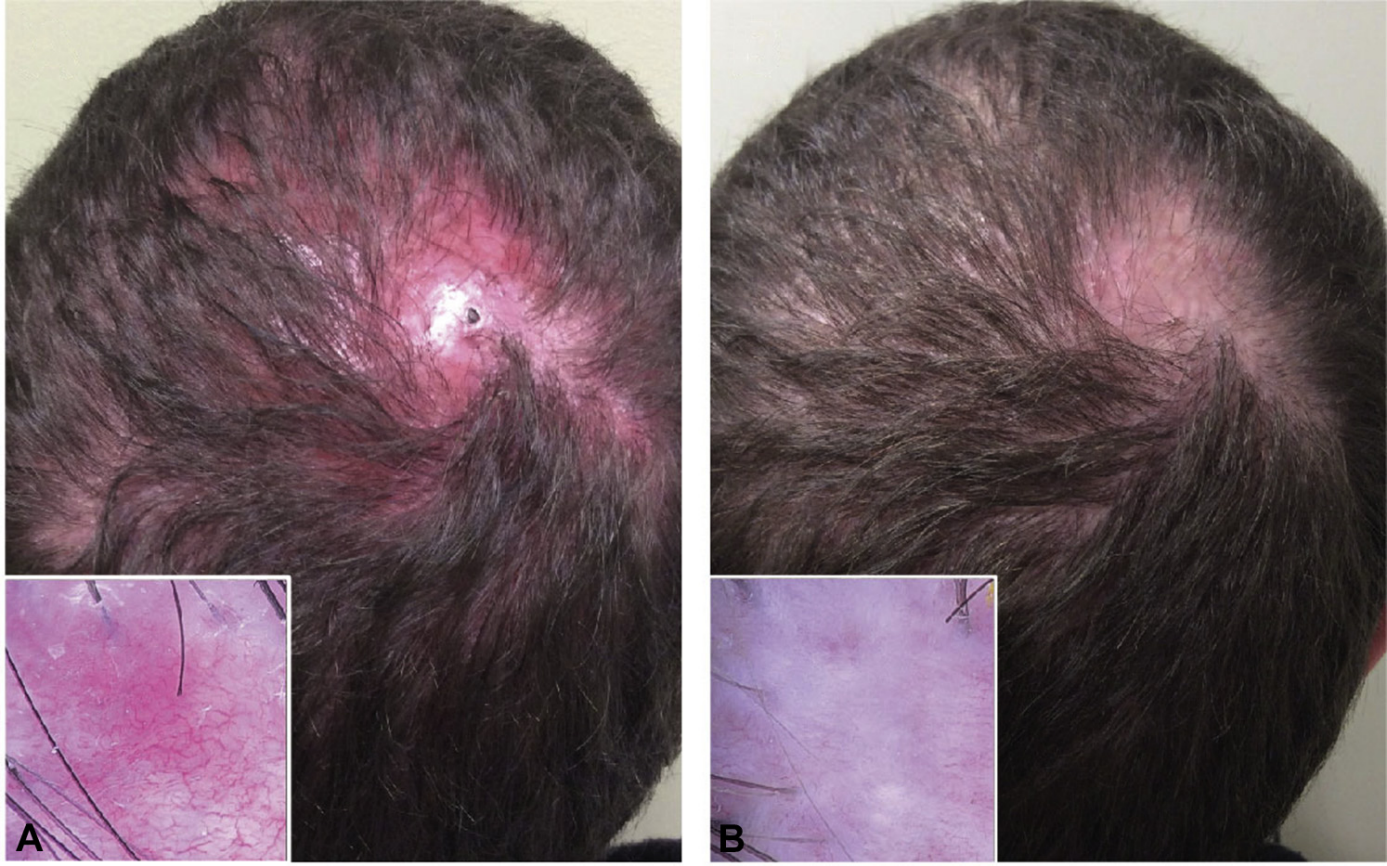
**A**, Baseline scalp photograph of the patient with folliculitis decalvans presented in case 1. **B**, After 4 platelet-rich plasma treatments. Insets show a zoomed-in view of the affected area using trichoscopy. (Original magnifications [of insets]: **A**, ×20; **B**, ×20.)

A 36-year-old man with a 4-year history of biopsy-proven FD was referred to our tertiary center for management. Clinical and trichoscopic evaluation revealed a scarred, alopecic patch at the vertex with perifollicular erythema (3+), scale (2+), and pustules (2+), accompanied by burning and pruritis (Fig 1, *A*). The patient was previously prescribed rifampin/clindamycin combination, doxycycline, ketoconazole 2% shampoo, and gentamicin 0.1% ointment, which led to an initial improvement for several months, followed by subsequent relapse and worsening of symptoms.

The patient was then treated for 6 months with oral isotretinoin 40 mg daily, intralesional triamcinolone (TAC), and topical clindamycin without any successful remission of symptoms. The therapy was discontinued as per his request because of insufficient symptom control. The patient chose to receive PRP treatments in combination with TAC at 5- to 6-week intervals and concomitant doxycycline 100 mg twice daily. Surprisingly, the patient reported symptomatic improvement after just the first PRP/TAC treatment, with symptom resolution. At the conclusion of 4 PRP treatments over 4 months, clinical and trichoscopic evaluation showed significantly reduced inflammation with reductions in perifollicular erythema (1+), scale (1+), and pustules (0). However, there was no change in hair density on the affected vertex (Fig 1, *B*). The patient self-reported a complete regression of itching and pain after each treatment, as well as stabilization of hair loss.

However, the clinical improvement was not sustained, as the patient reported disease flare within 4-5 weeks following PRP. To prolong the symptom-free interval between PRP treatments, we started oral apremilast 30 mg daily and oral hydroxychloroquine 400 mg daily. Currently, the patient is receiving PRP treatments at 8-week intervals, with good control of his FD for over 2 years.

## Case 2

**Fig 2:**
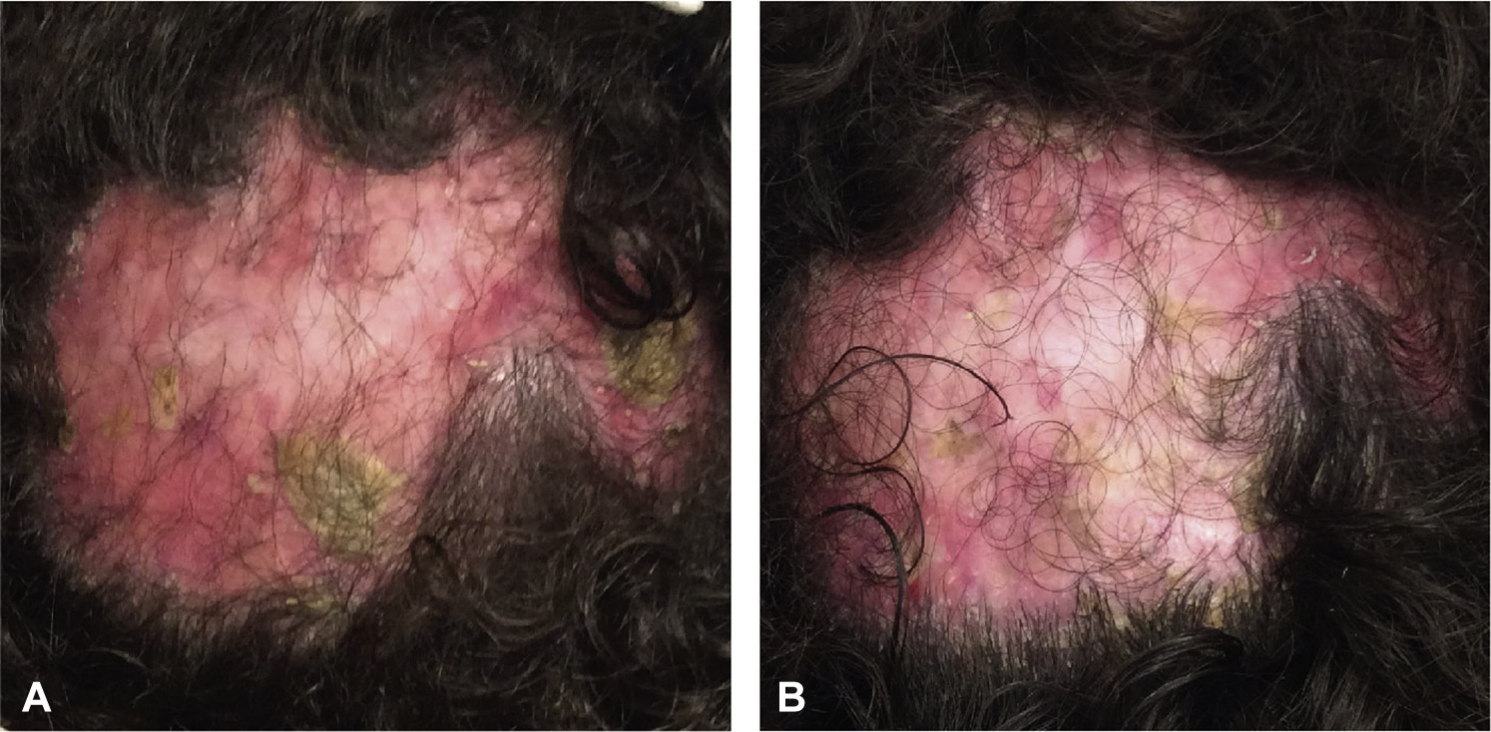
**A**, Baseline scalp photograph of the patient with folliculitis decalvans presented in case 2. **B**, After 3 platelet-rich plasma treatments.

A 25-year-old man with more than a 10-year history of biopsy-proven FD was referred to our tertiary center for management. Clinical examination revealed extensive scarring hair loss on the central scalp with perifollicular erythema (3+), scale (3+), pustules (3+), tufted hair, and purulent discharge (Fig 2, *A*). The patient had tried numerous treatments, including oral antibiotics (doxycycline, minocycline, clindamycin, rifampin, and trimethoprim-sulfamethoxazole), topical salicylic acid 5% shampoo, topical clindamycin lotion, oral isotretinoin 80 mg daily, and oral and intralesional corticosteroids for 6 months without any improvement. The patient received 3 PRP treatments in combination with TAC at 6- to 9-week intervals, while continuing oral doxycycline, topical salicylic acid shampoo, and topical clindamycin lotion. Upon completion of 3 PRP treatments, clinical examination showed a noticeable improvement in erythema (2+), scale (1+), and pustules (1+) and a resolution of purulent discharge (Fig 2, *B*). However, continued PRP treatment was necessary to maintain disease stability as the patient experienced a relapse within a 5-month break during COVID-19 quarantine.

## Discussion

There has been a growing interest in the use of PRP for treating scarring alopecias.^3^^,^^5^^,^^6^ To date, the efficacy of PRP in the treatment of neutrophilic scarring alopecia, such as FD, has not been studied. Here, we presented 2 cases of patients with refractory FD who had successful symptom control with PRP. Although the precise mechanisms underlying the efficacy of PRP in FD are unclear, it may be due to its anti-inflammatory and antimicrobial effects,^7^, ^8^, ^9^, ^10^ thus making it a promising therapy for many types of scarring alopecias.

Although the pathogenesis of FD is not fully understood, there is speculation that the disease reflects an abnormal immune response to scalp-associated microbes, particularly *Staphylococcus aureus*,^1^ leading to inflammation of the hair follicles. PRP could mitigate inflammation by releasing a high concentration of activated platelets and growth factors and increasing the secretion of anti-inflammatory cytokines such as interleukin 4, interleukin 10, interleukin 13, and transforming growth factor beta.^7^ PRP could also control microbial infections by causing a release of antimicrobial peptides known as platelet microbial proteins from activated platelets.^9^ These peptides have been shown to exert antimicrobial activity against a broad range of human pathogens, including *S aureus*.^8^ Additional evidence suggests that the activated platelets may mediate antimicrobial activity by generating oxygen metabolites that kill bacteria or by promoting the activation of monocytes and dendritic cells.^10^

The clinical improvements by PRP that we presented in 2 patients with refractory FD are encouraging, as there are few effective treatments for this physically and emotionally scarring disease. We noted that even a single PRP treatment significantly improved inflammation in both patients, in whom conventional FD therapies had failed. We acknowledge the limitations of our case series such as its small sample size and the possible confounding effects of other concomitant treatments. Additionally, the observed PRP effects were temporary and the disease relapsed in a time-dependent manner between treatment sessions in both cases. Therefore, it is important to conduct larger studies, including randomized placebo-controlled trials, to evaluate the extent of the clinical benefits of PRP in scarring alopecias, such as FD, over longer periods. It would also be valuable to investigate the relationship between different concentrations of growth factors in PRP and the clinical outcomes. As PRP can be a cost-prohibitive procedure, physicians should initiate shared decision-making discussions with their patients prior to initiating the therapy.

## Conflicts of interest

None disclosed.
